# Oral magnesium reduces levels of pathogenic autoantibodies and skin disease in murine lupus

**DOI:** 10.1186/s12865-024-00650-y

**Published:** 2024-09-06

**Authors:** Alberto Verlato, Teresina Laragione, Sofia Bin, Randie H. Kim, Fadi Salem, Percio S. Gulko, Paolo Cravedi

**Affiliations:** 1 Precision Immunology Institute, Translational Transplant Research Center, Icahn School of Medicine at Mount Sinai, One Gustave L Levy Place, Box 1243, New York, NY 10029 USA; 2 Renal Unit, Department of Medicine, University Hospital of Verona, Verona, Italy; 3 Division of Rheumatology, Department of Medicine, Icahn School of Medicine at Mount Sinai, One Gustave L Levy Place, Box 1243, New York, NY 10029 USA; 4 Nephrology, Dialysis and Renal Transplant Unit, IRCCS - Azienda Ospedaliero-Universitaria di Bologna, Bologna, Italy; 5 CIRI Scienze della Vita e Tecnologie per la Salute - Alma Mater Studiorum Università di Bologna, Bologna, Italy; 6 Dermatopathology Section, The Kimberley and Eric J. Waldman Department of Dermatology, Icahn School of Medicine at Mount Sinai, New York, NY USA; 7 Department of Laboratory Medicine and Pathology, Mayo Clinic, Jacksonville, FL USA

## Abstract

**Background:**

Systemic Lupus Erythematosus (SLE) has a strong genetic susceptibility, but little is known about the impact of diet on disease severity. The Western diet is typically deficient in magnesium (Mg), and given the immunomodulatory effects of Mg, we hypothesized that the low Mg intake increases disease risk and that increasing Mg intake would reduce severity of murine lupus. Here, we placed 12-week old MRL/lpr female lupus mice on a normal (Mg500) or a high (Mg2800) Mg diet for 9 weeks. Urine and blood were collected during the study for quantification of urinary albumin, BUN, anti-dsDNA antibodies, and immune phenotyping.

**Results:**

MRL/lpr lupus mice on high Mg2800 diet had significantly fewer skin lesions and less severe skin histology score, and reduced levels of pathogenic anti-dsDNA antibodies, compared with the Mg500 group (143.8±75.0 vs. 47.4±36.2 × 10^6^U/ml; *P* < 0.05). The high Mg2800 group had a nearly two-fold increase in the percentage of CD4^+^FOXP3^+^ Treg cells compared to controls (19.9±5.4 vs. 11.4±5.5%; *P* < 0.05). Treg percentages inversely correlated with the concentration of anti-dsDNA. None of the mice developed arthritis during the observation period and there were no significant differences in weight, proteinuria, BUN or kidney histology.

**Conclusion:**

In conclusion, oral supplementation of Mg has a protective effect in a murine lupus model and may represent an inexpensive and safe adjuvant in the treatment of SLE.

**Supplementary Information:**

The online version contains supplementary material available at 10.1186/s12865-024-00650-y.

## Introduction

Systemic Lupus Erythematosus (SLE) is a life-threatening autoimmune disease that affects skin, kidneys and other organs and is characterized by the production of pathogenic autoantibodies, including those targeting DNA [1]. SLE has a strong genetic susceptibility, and very little is known about its environmental and nutritional risk factors [1, 2] .

It has been estimated that the US diet is typically deficient in magnesium (Mg), with nearly 40% of the population consuming less than the recommended amounts [3]. Patients with SLE also commonly have low blood levels of Mg [4]. Magnesium has a central role in many intracellular functions [5, 6]. In vitro studies demonstrated that increased concentrations of Mg reduce LPS-induced production of pro-inflammatory cytokines such as TNF-α, IL-6 and IL-17 by monocytes and endothelial cells [7, 8], and these cytokines are increased in SLE and have been associated with disease activity [9, 10].

We have recently demonstrated that oral Mg supplementation reduces disease severity in two mouse models of rheumatoid arthritis by expanding FOXP3 + regulatory T cells (Treg) and IL-10 production through a microbiome-mediated mechanism [11]. Importantly, reduced Treg number and function has been described in SLE patients [12] and may be implicated in disease pathophysiology. Therefore, we postulated that the commonly Mg deficient US and Western diet favors pro-inflammatory pathways that contribute to SLE activity and/or severity. We tested this hypothesis administering a normal or a high Mg diet to mice that spontaneously develop SLE.

## Methods

### Mice

Female MRL/MpJ-Faslpr/J (MRL/lpr) mice were purchased from Jackson Laboratory (Bar Harbor, ME). At 12 weeks of age, 10 mice were started on a high Mg diet containing Mg2800 ppm and 10 mice were started on a regular diet (from here on referred as Mg500 ppm) as a control (see below for details). Both experimental and control groups were sacrificed at 21 weeks of age. Experiments were performed under an Institutional Animal Care and Use Committee (IACUC) approved protocol, and mice were maintained in the same room to limit potential effects of microbiome differences.

### Diet chow composition and regimens

***Dietary chow.*** The diets were purchased from Teklad-Envigo Laboratories (Somerset, NJ). Mice received identical diets, except for the amount of magnesium. Specifically, the diets were irradiated and had the following contents (g/kg): protein (17.7), carbohydrates (64.4), fat (6.2), casein (200), DL-methionine (3.0), sucrose (415), corn starch (250), soybean oil (60), cellulose (30), vitamin mix (Teklad 40060), ethoxyquin (antioxidant) (0.01), calcium phosphate, dibasic (13.7), potassium citrate (monohydrate) (7.7), calcium carbonate (4.8), sodium chloride (2.6), potassium sulfate (1.82), ferric citrate (0.25), manganous carbonate (0.12), zinc carbonate (0.056), chromium potassium sulfate (dodecahydrate) (0.02), cupric carbonate (0.012), potassium iodate (0.0004), and sodium selenite (pentahydrate) (0.0004).

The regular Mg diet had Mg oxide 0.822 g/kg of chow (Mg500 ppm) and the high Mg diet had Mg oxide 2.3 g/kg of chow (Mg2800 ppm). Twelve-week-old mice were randomly assigned to receive the normal Mg diet Mg500 or the high Mg diet Mg2800 for nine weeks.

The dose of high magnesium diet was based on our previous studies showing that a dose of magnesium nearly 5-fold the recommended daily requirements for mice was well-tolerated and induced significant reduction in arthritis severity and joint damage scores, while increasing number of FOXP3 + Tregs in arthritic C57BL/6 and DBA1/ mice [11].

### Clinical skin severity scoring

Inflammatory skin lesions on the dorsum of the neck, ears and forehead were scored based on a scale ranging from 0 to 3 as previously described [13]. 0 = no visible skin changes, 1 = minimal hair loss with redness and a few scattered lesions, 2 = redness, scabbing, and hair loss with a small area of involvement, and 3 = ulcerations with an extensive area of involvement.

### Arthritis activity and severity scoring

The clinical arthritis score was determined according to a scoring scale ranging from 0 to 16 per mouse per day as previously reported where 1 = swelling and erythema in a single joint, 2 = swelling and erythema in more than one joint, 3 = swelling of the entire paw and 4 = swelling of paw and inability to bear weight [14].

### Histological scoring of the skin and kidney lesions

Following euthanasia (100 mg/kg Ketamine/Xylazine by route of IP injection), mice were perfused with 4% paraformaldehyde in PBS at a rate of 8–10 ml/min. Skin and kidneys were paraffin-embedded (10%). Paraffin-embedded tissue sections (3 μm) were mounted on glass slides and stained with H&E (skin) or Periodic-Acid Schiff (PAS) (kidney). Light microscopy images were acquired on a wide-field microscope (Zeiss AxioImager Z2M). The histology slides were scanned and the high-resolution images scored blindly for treatment by two experienced pathologists (RK and FS, respectively) using digital imaging (WSI).

***Skin histology***: Skin histopathological lesions were graded from 0 to 2 for the following parameters: (a) degree of acanthosis, from 0 (none) to marked 2 (thickened dermis); (b) hyperkeratosis, 0 (none) to 2 (markedly increased keratin); inflammation, 0 (sparse) to 2 (heavy lymphocytic infiltrates); (c) fibrosis, dermal collagen, 0 (normal) to 2 (markedly thickened); (d) vessels, 0 (normal) to 2 (diffusely dilated); (e) ulcer, 0 (absent) or 1 (present). [15]

#### Renal histology

Kidney histopathological changes were quantitated based on previously reported scoring systems [15–17]. Glomerular involvement including mesangial proliferation, mesangial matrix hyperplasia and glomerulosclerosis, each was graded from 0 to 3 (0, absent; 1, mild; 2, moderate; 3, severe) with a maximum score of 9. Glomerular crescentic formation was graded from 0 to 3 (0, absent; 1, less than 25%; 2 more than 25%). The tubulo-interstitial compartment changes, including interstitial fibrosis, tubular atrophy and interstitial inflammation were each scored from 0 to 3 (0, absent; 1, in < 25% of the section; 2, in 25–50% of the section; 3, in 50–100% of the section) with a maximum score for 9. Vasculitis was reported if detected.

### Flow cytometry analysis

Spleens were harvested and individually analyzed. Single cell suspensions (1 to 3 × 10^6^) were stained with fluorescent-labelled monoclonal antibodies for cell surface antigens and incubated for 10 min at 25 °C, or 30 min at 4 °C. For intracellular staining cells were fixed with Cytofix/Cytoperm™ (Cat. 554714, BD Bioscience, San Jose, CA) for 20 min at 4 °C, followed by permeabilization in 1X Perm/Wash™ solution (Cat. 554723, BD Bioscience) for 15 min. In selected experiments, cells were fixed using eBioscience™ FOXP3/Transcription Factor Staining Buffer Set (Cat. 00-5523-00, Thermo Fisher Scientific, Waltham, MA). Fixed cells were stained with fluorochrome-conjugated antibodies for 30 min–1 h at 4 °C in the dark. CD4^+^FOXP3^+^ regulatory T cells (Tregs) were stained using anti-CD4-PE-Cy7 (clone GK1.5) (TONBO bioscience, San Diego, CA), anti-CD8a-Pacific Blue (clone 53 − 6.7) (Biolegend, San Diego, CA), and anti-FOXP3-APC (clone FJK-16 S) (Thermo Fisher Scientific). Tr1 cells were identified by anti-CD4-Pacific blue (clone: RM4-5), anti-CD45RA-PE (clone: 14.8), anti-CD49B-PeCy7 (clone: DX-5), anti-LAG3-APC (clone: C9B7W) and anti-IL-10-PerCP/Cy5.5 (clone: JES5-16E3). T_FH_ cells were stained with anti-CD4-Pacific blue (clone: RM4-5), anti-CXCR5-biotin-APC (clone 2G8), anti-PD1-PeCy7 (clone: RMP1-30), anti-BCL6-PE (Clone: IG191E/A8) and anti-IL-10-PerCP/Cy5.5 (clone: JES5-16E3) (antibodies from Biolegend or BD Bioscience). In selected experiment T_FH_ cells were analyzed using biotinylated anti-CXCR5 (clone 2G8) (BD Pharmingen) followed by Pacific Blue streptavidin (Thermo Fisher Scientific), anti-TCRb-PerCP-Cy5.5 (clone H57-597), anti-PD1-PE-Cy7 (clone RMP1-30), anti-CD4-BV510 (clone GK1.5) (BD Pharmingen), and anti-FOXP3-FITC (clone FJK-16 S) (Thermo Fisher Scientific). Germinal Center and Class switched B cells were stained using anti-B220-Pacific Blue (clone RA 3–6 B2), anti-FAS-APC (clone Jo2), and anti-GL7-FITC (clone GL7) (BD Pharmingen); anti-IgD-APC-Cy7 (clone 11-26c), and anti-IgM-PE-Cy7 (clone eB121-15F9) (Thermo Fisher Scientific).

For intracellular cytokine analysis, cells were treated with GolgiPlug (1 µg/ml; BD Biosciences, at 37 °C for 4 h), and stained with ) (BioLegend), anti-CD4-APC-Cy7 (clone GK1.5) (TONBO bioscience), anti-CD8-PacBlue (clone 53 − 6.7) (Biolegend), anti-IL-1B-: PE-Cy7 (clone NJTEN3 ) (company eBioscience), anti-TNF-α-FITC (clone MP6-XT22) (eBioscience), and anti-INF-ɣ-APC (clone XMG1.2) (eBioscience). At least 50,000 cells were acquired per sample. Samples were acquired on a BD LSRII, on a three-laser Canto II (BD Biosciences) flow cytometer, and analyzed with FlowJo (https://www.flowjo.com) software (Ashland, OR).

### Anti-dsDNA antibodies

Blood was collected via puncture of the submandibular vein, and serum isolated for ELISA. Antibodies were quantified using a commercially available kit (Mouse anti-dsDNA IgG-specific ELISA Kit, Cat. 5120, Alpha Diagnostic Intl. Inc., San Antonio, TX).

### Urinary and serum renal function chemistry measurements

Urine samples were collected from individual mice through gentle restrain in a collection device. Urine creatinine and albumin were quantified using commercial kits (Cayman Chemical, Cat. 500701, Ann Harbor, MI; Bethyl Laboratory Inc., Cat. E99-134, Montgomery, TX, respectively). Albuminuria was expressed as the ratio of urine albumin to urine creatinine. Serum BUN was quantified in serial blood collections using a colorimetric detection kit (Thermo Scientific, Cat. EIABUN) according to the manufacturers’ instructions.

### Serum levels of cytokines/chemokines

Blood samples were collected after nine weeks on the Mg500 or Mg2800 diets, and serum used for cytokine quantification. BAFF, IFN-ɣ, IL-6, IL-10, IL-16, IL-18, IL-21, IP-10 (CXCL10) and TNF-α were measured using a bead-based multiplex array ProcartaPlex mouse Mix&Match panel (ThemoFisher Scientific). All specimens were assayed in duplicate according to the manufacturer’s instructions. The assay was performed using MAGPIX instrument with xPONENT software (Luminex Corporation, Austin, TX) and analyte concentration calculated from standard curves using 5-parameter logistic curve fit.

### Statistics

All variables were normally distributed and therefore means were compared with unpaired t-test or two-way ANOVA (Sidak’s multiple comparison test). P values < 0.05 were considered significant. All statistical analyses were performed using GraphPad Prism (version 8 for Windows, GraphPad Software, Inc.).

## Results

### High Mg diet (Mg2800) reduces the skin disease severity in MRL/lpr lupus mice

**Fig. 1:**
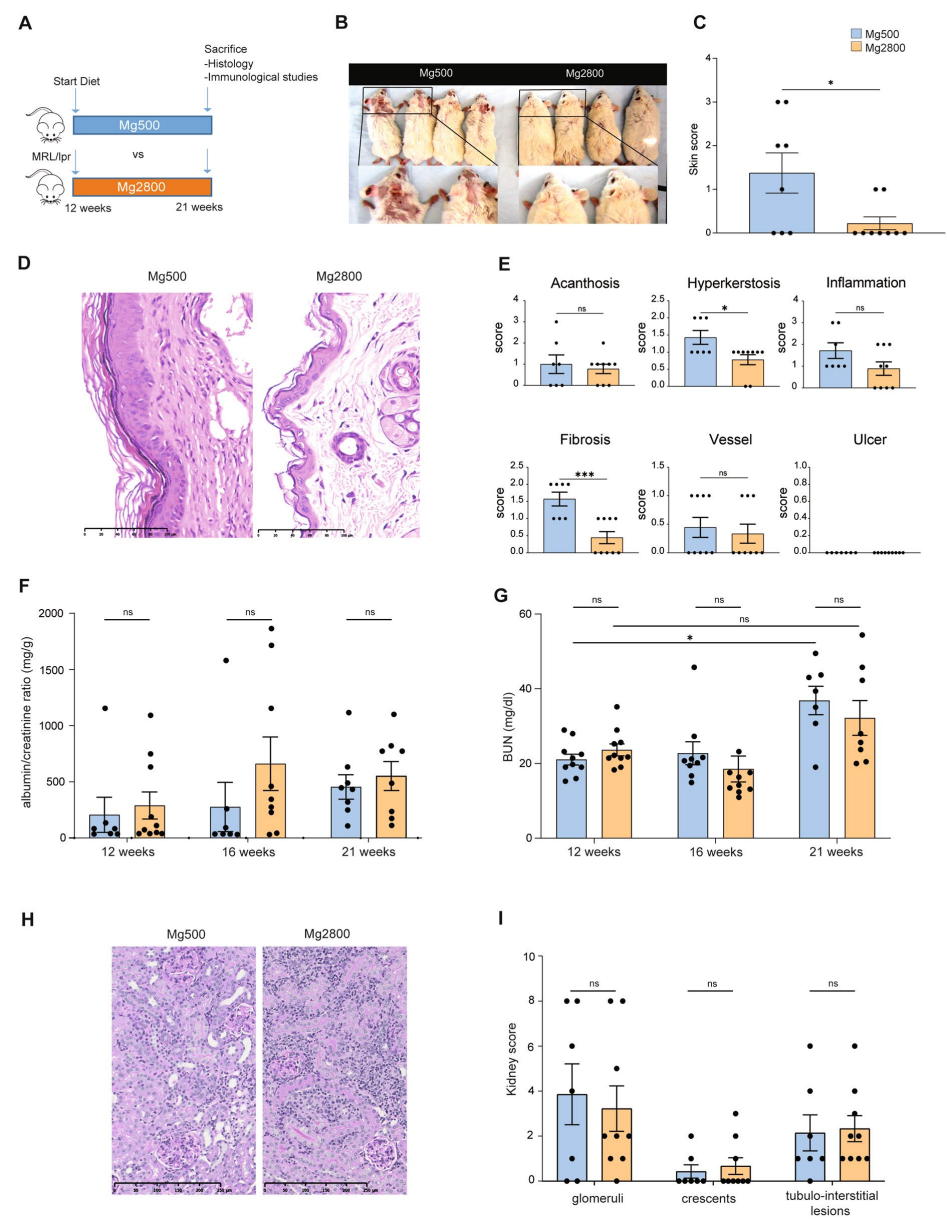
High Mg diet reduces disease severity in murine lupus. **A**) Study design; **B**) representative pictures of skin lesions with insert showing a magnified view; **C**) skin clinical lesion scores at sacrifice; **D**) representative pictures of histological skin lesions (H&E staining; size bar is included); **E**) histological skin lesion scoring; **F**) changes in urinary albumin over creatinine ration; **G**) BUN in the high and normal Mg diet groups; **H**) representative pictures of kidney histological lesions (H&E staining; size bar is included) and **I**) histological scoring of the kidney sections. Each dot represents a separate mouse; *P < 0.05; ***P < 0.001; ns: not significant. Data in bar graphs represent mean ± S.E.M

MRL/lpr mice received either a high (Mg2800) or regular (Mg500) Mg diet from week 12 to week 21 of age (Fig. 1A). The diet was well-tolerated, and mice did not have diarrhea or weight loss, with similar weights on both groups (Supplementary Fig. 1). During the treatment period, 4 mice died (3 mice in the Mg 500 and 1 in the Mg 2800 group). We did not ascertain the cause of death.

Mice on high Mg diet had significantly lower clinical skin severity score than controls (21 weeks; Fig. 1B and C). Consistently, histology analyses of skin lesions also revealed significant protection in the high Mg diet group, with lower hyperkeratosis and fibrosis scores compared with the normal Mg diet group (Fig. 1D-E). Skin inflammation scores also tended to be lower in the high Mg group, but that difference did not reach statistical significance (Fig. 1E).

None of the mice developed arthritis during the nine weeks of observation. Both treatment groups developed similar levels of albuminuria (Fig. 1F) and similar kidney failure (Fig. 1G). Renal pathology scores were also similar in both treatment groups (Fig. 1H-I**)**.

### High Mg diet reduces levels of pathogenic anti-dsDNA autoantibodies

**Fig. 2:**
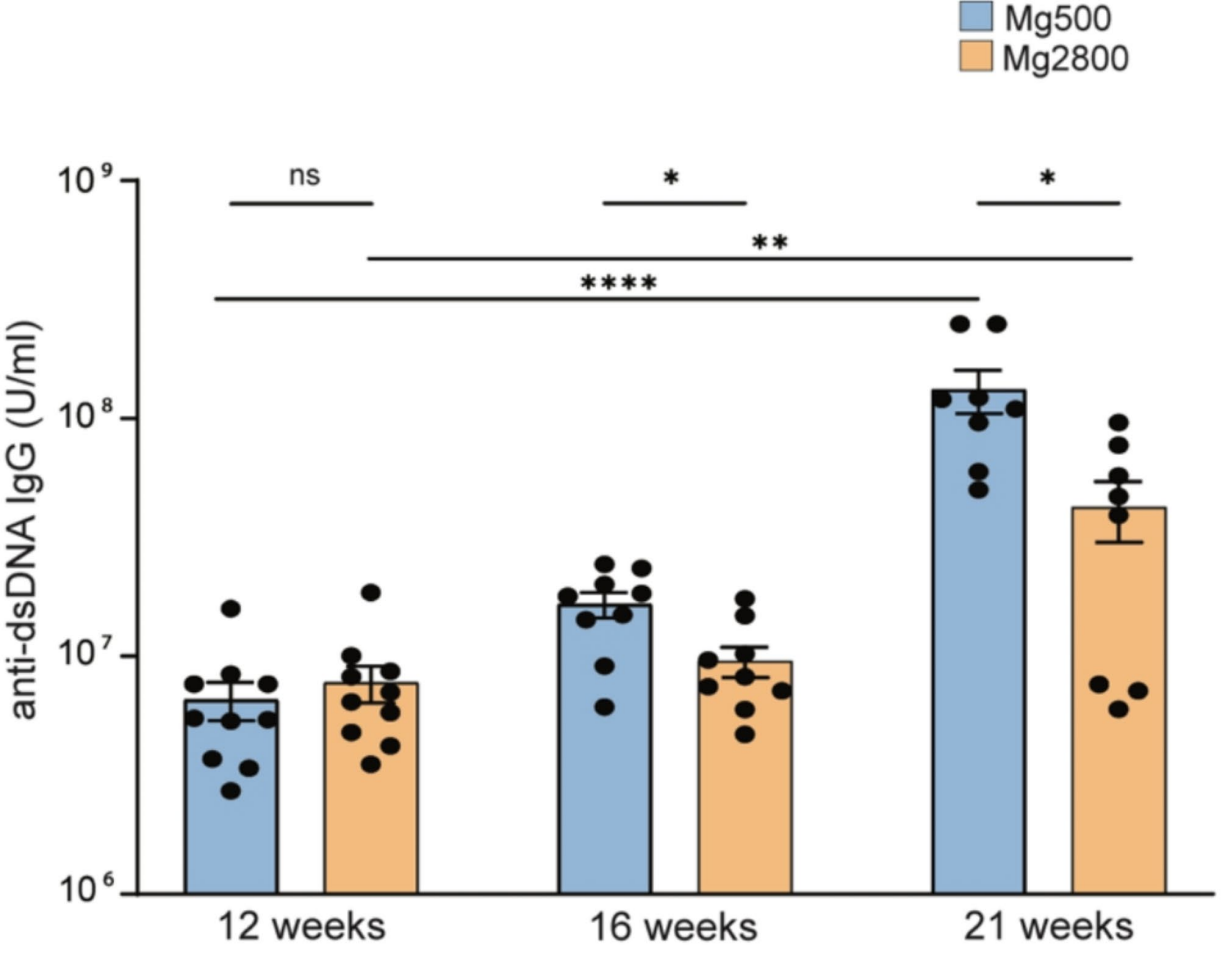
High Mg diet reduces levels of pathogenic anti-dsDNA autoantibodies in murine lupus. Anti-dsDNA IgG levels in the high and normal Mg diet groups at serial time points after diet initiation. Each dot represents a separate mouse; *P < 0.05. **P < 0.01; ****P < 0.0001; ns: not significant. Data in bar graphs represent mean ± S.E.M

The levels of IgG anti-dsDNA progressively increased during the disease course in both groups. However, the levels were significantly lower in mice on the high Mg diet group at both 16 and 21 weeks of age, compared to the normal diet controls (Fig. 2).

### The high Mg diet increases numbers of splenic CD4+CD25+FOXP3+ Treg cells

**Fig. 3:**
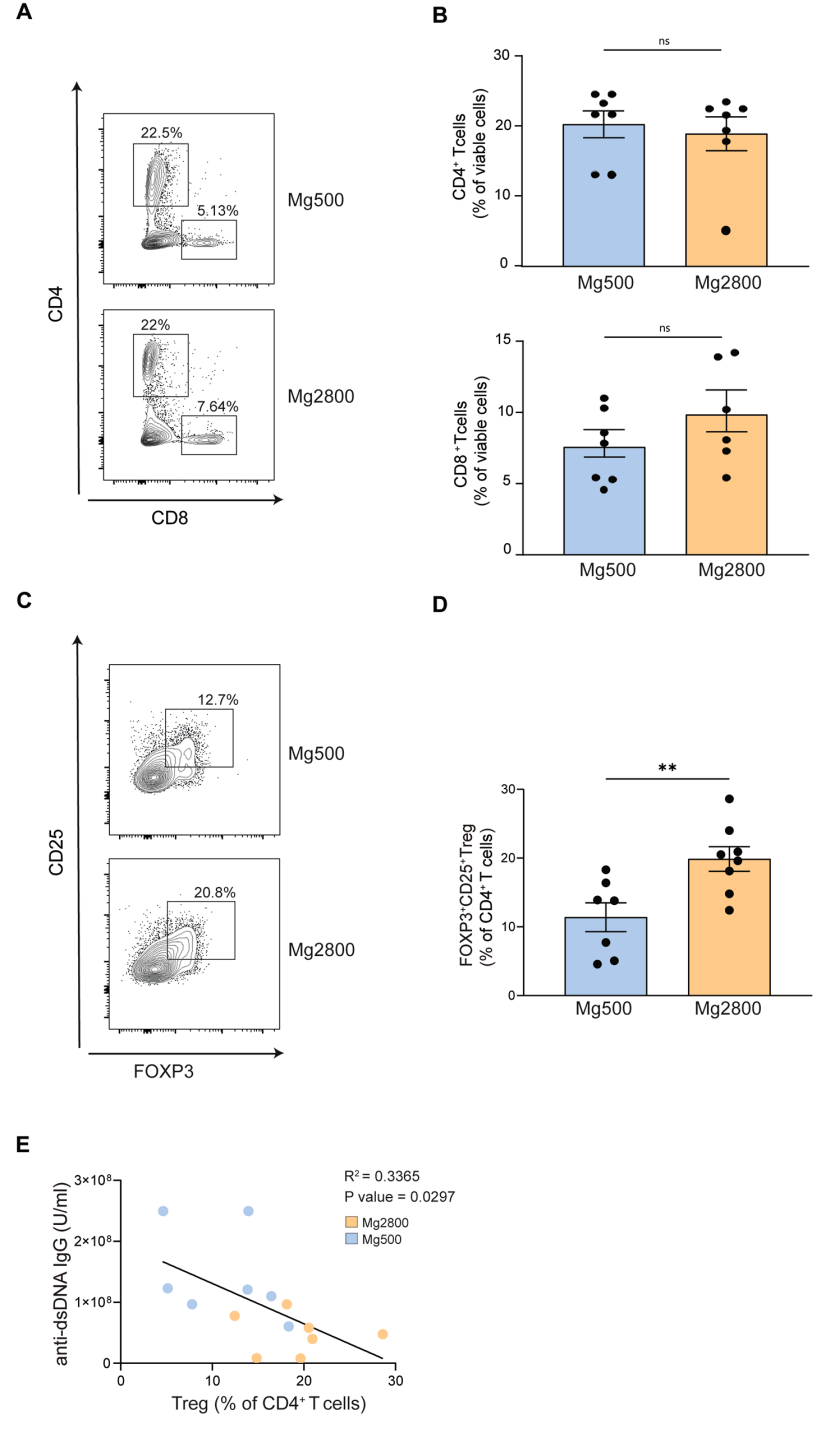
High Mg diet increases regulatory T cells. **A**) Representative plots and **B**) data quantification of splenic CD4 + and CD8 + T cells in the high and normal Mg diet groups. **C**) Representative plots and **D**) data quantification of splenic CD4^+^CD25^+^FOXP3^+^ Treg in the high and normal Mg diet groups at sacrifice (21 weeks of age); **E**) Correlation between percentages of splenic Treg cells and levels of circulating anti-dsDNA IgG in the high and normal Mg diet groups. Each dot represents a separate mouse; **P < 0.01. Data in bar graphs represent mean ± S.E.M

The immune phenotype of splenocytes was analyzed at week 21. No significant differences were observed in the percentages of CD4^+^ or CD8^+^ T cells (Fig. 3A-B). Percentages of splenic CD4^+^CD25^+^FOXP3^+^ Treg were significantly higher in the high Mg diet group, compared to the normal Mg group (Fig. 3C-D). Importantly, Treg percentages inversely correlated with the concentrations of anti-dsDNA IgG (Fig. 3E).

There were no significant differences in the percentages of T follicular helper (T_FH_), T follicular regulatory T cells (T_FR_), germinal center B cells, or memory B cells between high and normal Mg diet groups (Supplementary Fig. 2). We next examined intracellular levels of the pro-inflammatory cytokines IL-1β, IFN-γ, and TNF-α in CD4^+^ and CD8^+^ T cells, but detected no significant difference between the two experimental groups (Supplementary Fig. 3).

### Serum cytokines

We quantified the serum levels of nine different cytokines implicated in lupus pathogenesis, but we found no significant difference in seven of them (Supplementary Fig. 4). IL-16 levels tended to be lower in the high Mg group, but that difference did not reach statistical significance. IL-10 and IL-21 were below the detection level in most of the mice.

## Discussion

SLE is a potentially life-threatening autoimmune disease with strong genetic and environmental contributions to disease susceptibility and severity [1]. Yet, little is known about the dietary risk factors for SLE [2]. In the present study, we report that a high Mg diet (Mg2800) reduced murine SLE skin disease severity and levels of pathogenic anti-dsDNA autoantibodies. The high Mg diet also increased numbers of CD4^+^FOXP3^+^ Tregs, and FOXP3^+^ Treg cells can reduce autoreactive B cell responses and the production of pathogenic anti-dsDNA autoantibodies [18]. We observed a similar effect where Treg cells inversely correlated with anti-dsDNA IgG levels, suggesting a mechanistic link between increased Treg and reduced autoantibodies in the Mg2800 diet mice. Numbers of CD4^+^FOXP3^+^ Tregs are typically reduced in human and murine SLE, as well as in other autoimmune diseases [19–21], and our findings suggest a potential new role for dietary Mg supplementation in the treatment of SLE.

Treg cells can be expanded by low-dose IL-2 [22], and by products of the intestinal microbiome [23]. We have recently shown that a high Mg diet expands Tregs and reduces experimental arthritis through an intestinal microbiome-dependent mechanism [11]. High Mg diet increased the levels of Bacteroides and other bacteria associated with increased production of short-chain fatty acids (SCFA) that have been implicated in the regulation of immune cells [24–26]. Therefore, we speculate that similar mechanisms take place in lupus mice receiving a high Mg diet. Specifically, we considered that increased Mg intake modifies the intestinal microbiome expanding the representation of SCFA-producing bacteria to increase the numbers of Treg cells hypothesize that similar effects may be achieved in patients. We previously showed that the high Mg diet increases IL-10-producing Tr1 cells [11], a T cell subset that plays an important role in maintenance of immunological tolerance [27]. However, we did not observe an increase in Tr1 cells in MRL/lpr mice on high-Mg diet, raising the possibility that the pro-inflammatory environment of this model may prevent some of the protolerogenic effects of Mg to be fully recapitulated, or perhaps a strain-specific effect as the intestinal microbiome may differ between strains.

We did not include a low Mg diet group in this study given that it is a poorly tolerated diet and is associated with increased mortality [28–30], and therefore would not be a feasible therapeutic option for patients.

The environmental and nutritional risk factors for SLE are just beginning to be unveiled [2, 31]. Identifying new modifiable nutritional and environmental risk factors has the potential to significantly impact disease prevention, and the treatment of SLE patients.Our observations suggest that Mg supplementation has the potential to be beneficial even in the absence of a Mg deficiency or inadequate intake. Oral Mg supplementation is a benign, inexpensive and typically safe and well-tolerated over the counter supplement. Our study provides the basis for a future study aimed at testing the hypothesis that Mg supplementation reduces disease severity in patients with SLE and determining whether the same effects are observed in levels of anti-DNA antibodies and Tregs. It will also be interesting to characterize the effects of the Mg supplementation in the intestinal microbiome of SLE patients.

In conclusion, we describe for the first time that increasing Mg intake can significantly ameliorate skin disease severity in a murine model of SLE, and show that it expands Tregs and reduces pathogenic anti-dsDNA autoantibodies.

## Electronic supplementary material

Below is the link to the electronic supplementary material.

Supplementary Material 1

## Data Availability

Data will be available upon request to the corresponding authors.

## Declarations

### Author information

Drs. Verlato, Laragione, and Bin were involved in the experiments with mice. Drs. Kim and Salem scored the severity of skin and kidney histological lesions, respectively. All authors were involved in drafting the article or revising it critically for important intellectual content, and all authors approved the final version of this manuscript. Drs. Cravedi and Gulko had full access to all of the data.

## Abbreviations

Anti-dsDNA: Anti-double stranded DNA
Mg: Magnesium
Treg: Regulatory T cell
SLE: Systemic Lupus Erythematosus
T_FH_: T follicular helper cell
T_FR_: T follicular regulatory T cell
