# Correction: Oral Magnesium reduces levels of pathogenic autoantibodies and skin disease in murine lupus

**DOI:** 10.1186/s12865-024-00658-4

**Published:** 2024-10-10

**Authors:** Alberto Verlato, Teresina Laragione, Sofia Bin, Randie H. Kim, Fadi Salem, Percio S. Gulko, Paolo Cravedi

**Affiliations:** 1 Precision Immunology Institute, Translational Transplant Research Center, Icahn School of Medicine at Mount Sinai, One Gustave L Levy Place, Box 1243, New York, NY 10029 USA; 2 Renal Unit, Department of Medicine, University Hospital of Verona, Verona, Italy; 3 Division of Rheumatology, Department of Medicine, Icahn School of Medicine at Mount Sinai, One Gustave L Levy Place, Box 1243, New York, NY 10029 USA; 4 Nephrology, Dialysis and Renal Transplant Unit, IRCCS - Azienda Ospedaliero-Universitaria di Bologna, Bologna, Italy; 5 CIRI Scienze della Vita e Tecnologie per la Salute - Alma Mater Studiorum Università di Bologna, Bologna, Italy; 6 Dermatopathology Section, The Kimberley and Eric J. Waldman Department of Dermatology, Icahn School of Medicine at Mount Sinai, New York, NY USA; 7 Department of Laboratory Medicine and Pathology, Mayo Clinic, Jacksonville, FL USA

**Correction: BMC Immunology (2024) 25:58**

10.1186/s12865-024-00650-y

Following publication of the original article [1], the title was incorrectly given as ‘Revised version with tracked changes oral Magnesium reduces levels of pathogenic autoantibodies and skin disease in murine lupus’ but should have been ‘Oral Magnesium reduces levels of pathogenic autoantibodies and skin disease in murine lupus’.

The original article has been corrected.
