# Supplementary material for: Oral magnesium reduces levels of pathogenic autoantibodies and skin disease in murine lupus
Source: BMC Immunol. 2024 Sep 6;25:58. doi: 10.1186/s12865-024-00650-y (PMC11378425; doi:10.1186/s12865-024-00650-y)
Supplement: Supplementary file 1 — Supplementary Material 1 [file 12865_2024_650_MOESM1_ESM.docx]

**Oral Magnesium reduces levels of pathogenic autoantibodies and skin disease in murine lupus.**

**Supplementary Material**

Contents:

1. Figure S1. Weight at serial time points after normal (Mg500) or high (Mg2800) diet initiation.
2. Figure S2. Percentages of Tr1, T follicular, and B cells at the end of the dietary treatment in the Mg500 and Mg2800 groups.
3. Figure S3. Percentages of CD4 and CD8 intracellular cytokines.
4. Figure S4. Serum levels of cytokines at the end of the dietary treatment in the Mg500 and Mg2800 groups.

Figure S1. Weight at serial time points after normal (Mg500) or high (Mg2800) diet initiation. Each dot represents a separate mouse. Data in bar graphs represent mean ± S.E.M.

Figure S2. Percentages of Tr1, T follicular, and B cells at the end of the dietary treatment in the Mg500 and Mg2800 groups. A) T regulatory 1 cells (Tr1), B) T follicular helper cells (Tfh) and C) T follicular regulatory cells (Tfr). Panels D and E show percentages of germinal center (GC) and memory (Mem) B cells. Each dot represents a separate mouse. Data in bar graphs represent mean ± S.E.M.

Figure S3. Percentages of CD4 and CD8 intracellular cytokines. intracellular IL1β+, IFNγ+, and TNFα+ A) CD4+ and B) CD8+ T cells in the Mg500 and Mg2800 groups. Each dot represents a separate mouse. Data in bar graphs represent mean ± S.E.M.

Figure S4. Serum levels of cytokines at the end of the dietary treatment in the Mg500 and Mg2800 groups. Each dot represents a separate mouse. Data in bar graphs represent mean ± S.E.M.
